# Chaos and multi-scroll attractors in RCL-shunted junction coupled Jerk circuit connected by memristor

**DOI:** 10.1371/journal.pone.0191120

**Published:** 2018-01-17

**Authors:** Jun Ma, Ping Zhou, Bashir Ahmad, Guodong Ren, Chunni Wang

**Affiliations:** 1 School of Science, Chongqing University of Posts and Telecommunications, Chongqing, China; 2 Department of Physics, Lanzhou University of Technology, Lanzhou, China; 3 NAAM-Research Group, Department of Mathematics, King Abdulaziz University, Jeddah, Saudi Arabia; Beijing University of Posts and Telecommunications, CHINA

## Abstract

In this paper, a new four-variable dynamical system is proposed to set chaotic circuit composed of memristor and Josephson junction, and the dependence of chaotic behaviors on nonlinearity is investigated. A magnetic flux-controlled memristor is used to couple with the RCL-shunted junction circuit, and the dynamical behaviors can be modulated by changing the coupling intensity between the memristor and the RCL-shunted junction. Bifurcation diagram and Lyapunov exponent are calculated to confirm the emergence of chaos in the improved dynamical system. The outputs and dynamical behaviors can be controlled by the initial setting and external stimulus as well. As a result, chaos can be suppressed and spiking occurs in the sampled outputs under negative feedback, while applying positive feedback type via memristor can be effective to trigger chaos. Furthermore, it is found that the number of multi-attractors in the Jerk circuit can be modulated when memristor coupling is applied on the circuit. These results indicate that memristor coupling can be effective to control chaotic circuits and it is also useful to reproduce dynamical behaviors for neuronal activities.

## Introduction

Chaos emergence can be observed in complex nonlinear systems [1–3]. In a practical way, nonlinear circuits [4–7] can generate chaotic series by setting appropriate parameters carefully, and it is believed that chaotic systems can be used for secure communication and image encryption [8–12]. Within the dimensionless dynamical models, nonlinearity is necessary for generating chaos and hyperchaos that one positive Lyapunov exponent at least can be detected in the sampled times for observable variables. Chaotic behaviors are often regarded as harmful and chaos should be suppressed; therefore, many schemes have been proposed to control the chaos [13–17]. Particularly, the author of this paper calculated the Hamilton energy [18] of dynamical system and suggested that energy modulation [19] can be used to control the dynamical states of hidden attractors. On the other hand, to keep consensus and realize synchronization, some chaotic systems are often used to investigate different kinds of synchronization problems. For example, parameter estimation [20–23] based on synchronization is discussed, and thus self-adaptive control schemes are appreciated for reaching synchronization and control [24–27] on chaotic systems. In the case of chaos suppression and stabilization, uncertainty and control period should be considered. For example, *Mobayen et al*.[28,29] discussed the finite‐time stabilization and second-order fast terminal sliding mode control design for chaotic systems with uncertainty, and the reliability of LMI approach has been confirmed. Furthermore, this scheme is carried out on synchronization problems even associated with fractional order dynamical system as well [30,31]. Indeed, the synchronization transition and control [32–35] on chaotic networks become more attractive because spatiotemporal systems show more complex dynamical behaviors than low-dimensional chaotic systems. All the nodes of the network used to keep pace with each other and the network becomes homogeneous state when complete synchronization is reached. Indeed, pattern formation and selection [36–42] could be another interesting aspect to investigate the collective behaviors of network. For example, spiral waves and Turing patterns can be induced and developed in biological networks, ecological systems and square array composed of coupled oscillators [43,44]. In fact, the self-organization and synchronization behaviors mainly depend on the local kinetics of nodes. Therefore, it is important to investigate the dynamics of isolated oscillators with different nonlinear properties, and it could be helpful for further investigation on collective behaviors of coupled oscillators and networks.

To enhance the complexity and nonlinearity, time delay is often introduced into some dynamical systems. For isolated oscillators, time delay can be thought as intrinsic, and it is often called as response time delay. For some interneurons, autapse[45,46], which is a specific synapse connects the axon and dendron or soma, introduces time delay along the close loop. As a result, the self-adaption of neuron to external forcing and stimulus is enhanced [47–49]. For example, appropriate distribution of autapse in the neuronal network can enhance the synchronization degree [50], and regular spatial patterns [51,52] can be developed. As argued by the author in Ref. [53], it is believed that the autapse formation could be associated with the injury on axon of neuron, thus an auxiliary loop is set up to help signal propagation and overcome the injured area. In setting of chaotic circuits, nonlinear electric devices such as negative resistor, nonlinear capacitor, and nonlinear inductor are critical to supply nonlinearity of the circuits. Josephson junction [54,55] is an important nonlinear inductor because of its sensitivity and superconductivity, and it plays important role in constructing chaotic circuits. For example, Josephson junction coupled resonator is used to simulate the electrical activities of neuron [53,56,57]. Particularly, a new electric device memristor [44,58,59], whose memductance is dependent on the inputs current, is often used to design various chaotic systems [60–62] for dynamical analysis and potential application for signal processing. It is confirmed that memristor-based circuit can show distinct memory effect because the memductance is fixed when external stimulus on memristor is moved. Due to its memory effect, the author of the paper suggested that memristor coupling and magnetic flux [63] can be used to describe the effect of electromagnetic induction and radiation in electrical activities of neurons [64–66]. Furthermore, memristor coupling is used to model the transition of electrical activities in cardiac tissue exposed to electromagnetic radiation, and the potential mechanism of heart disease induced by electromagnetic radiation is explained [67]. The improved neuronal circuits composed of memristors are used to investigate the synchronization behaviors [68], while extensive potential application of memristor keeps open.

In this paper, memristor is used to bridge a RCL-shunted junction circuit and the jerk circuit composed of infinite scroll attractors, thus an extended chaotic system can be formed for dynamical analysis and control. By changing the feedback gain in memristor on the coupled circuit, the infinite scroll attractors can be stabilized, and chaos can also be controlled.

## Model setting and description

**Fig 1:**
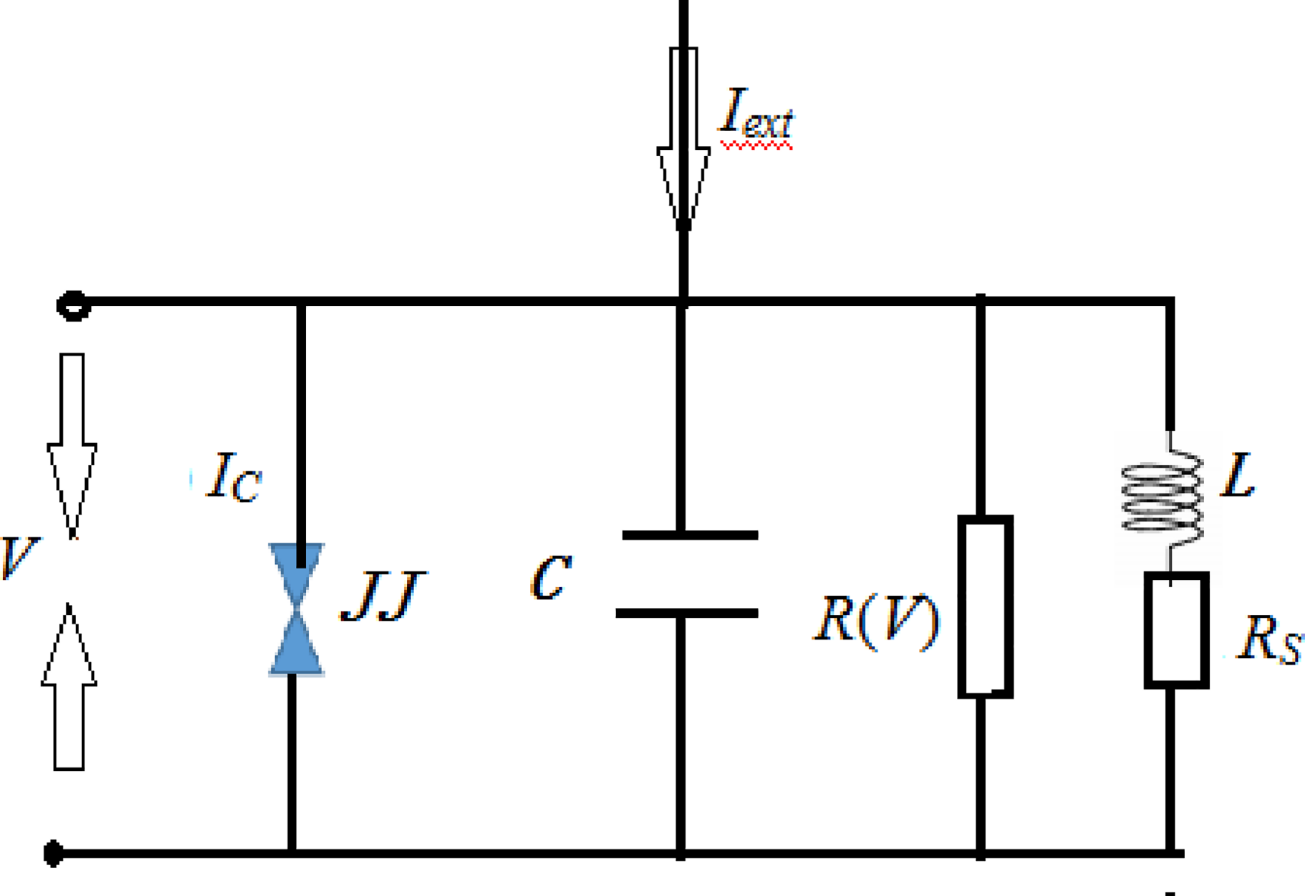
Diagram for RCL-shunted junction circuit. A Josephson junction is represented by three parallel channels, the supercurrent *I*_*c*_*sin*(*γ*), a capacitor *C* due to the overlap geometry, and a resistive channel modeling the quasiparticle leakage current. An inductance *L* followed with a shunt resistance *R*_*s*_ is used as a shunt branch.

Due to the sensitive effect of electrical inductance, Josephson junction is often used to couple with resistor, capacitor, and inductor. As a result, a RCL-shunted circuit can be designed for potential analysis and synchronization application [69,70]. The circuit diagram is plotted in Fig 1.

According to the Kirchhoff's law, the circuit equations can be described as follows

$$\left\{ \begin{array}{l} C\frac{dV}{dt}=I_{ext}-\frac{V}{R(V)}-I_{c}\sin(\gamma )-I_{s}-I_{N}\\ \frac{\hbar }{2e}\frac{d\gamma }{dt}=V\\ L\frac{dI_{s}}{dt}=V-I_{s}R_{s}-V_{N} \end{array}\right.;$$ (1)

where *I*_*s*_ denotes the current flowing in the shunt branch, *L* and *R*_*s*_ is the shunt inductance and shunt resistance, respectively. *I*_*N*_, *V*_*N*_ is the fluctuating current noise and voltage noise that are presented in the dissipative elements *R(V)* and *R*_*s*_, respectively. The parameter *ħ* represents Planck's constant, and *C* is the capacitance. As reported in Ref.[71], a voltage-dependent junction resistance is defined by

$$R(V)=\left\{ \begin{array}{ll} R_{N} & if\left|V\right|>V_{g}\\ R_{sg} & if\left|V\right|\leq V_{g} \end{array}\right.$$ (2)

A scale transformation is applied on Eq (1) to find a dimensionless dynamical system by using the following criterion.

$$\left\{ \begin{array}{l} \tau =\omega_{c}t=t2eI_{c}R_{s}/\hbar ;\beta_{c}=2eI_{c}R^{2}{}_{s}C/\hbar ;\beta_{L}=2eI_{c}L/\hbar ;i=I_{ext}/I_{C};g=R_{s}/R(V);\\ x_{1}=\gamma \;\mathrm{mod}\;2\pi ;x_{2}=V/I_{c}R_{s};x_{3}=I_{s}/I_{c}; \end{array}\right.$$ (3)

When the noise terms *I*_*N*_, *V*_*N*_ are neglected, a shunted linear resistive-capacitive junction (RCLSJ) [70, 72] is described by

$$\left\{ \begin{array}{l} {\dot{x}}_{1}=x_{2}\\ {\dot{x}}_{2}=(i-g(x_{2})x_{2}-\sin x_{1}-x_{3})/\beta_{C}\\ {\dot{x}}_{3}=(x_{2}-x_{3})/\beta_{L} \end{array}\right.;$$ (4)

$$g(x_{2})=\left\{ \begin{array}{ll} 0.366 & \left|x_{2}\right|>2.9\\ 0.061 & \left|x_{2}\right|\leq 2.9 \end{array}\right.;$$ (5)

where the variable *x*_1_, *x*_2_, and *x*_3_ often denotes the phase difference, junction voltage and current through the shunted inductance, respectively. The nonlinear function *g*(*x*_2_) measures the current-voltage relation between junction resistances in Eq (2). The parameter *β*_C_, *β*_L_, defines the capacitive, inductance constants, respectively. For more detailed description, readers can refer to Ref.[71] and references therein. As confirmed in Ref.[73], chaotic state can be detected when the parameters are fixed at *β*_C_ = 0.707, *β*_L_ = 2.6, and external forcing DC current *i* = 1.2. In fact, infinite equilibrium points are approached as (*nπ*, 0, 0) when external forcing current is removed, where *n* is integer and *n* = 1, 2, 3, …*N*. Surely, bifurcation and linear stability analysis [74] could also be helpful to confirm the emergence of chaos in Josephson junction-coupled system. To enhance the nonlinearity and complexity, memirstor [75,76] is used for setting circuits which the outputs much depend on the initial setting for variables. For example, *Chua et al*. [76] presented a rigorous and comprehensive nonlinear circuit-theoretic foundation for the memristive Hodgkin-Huxley axon circuit model, which comprised a potassium ion-channel memristor and a sodium ion-channel memristor, along with some mundane circuit elements. It is known that the memductance of memristor is dependent on the inputs current, and the magnetic flux-dependent memristor is described by

$$\rho (\varphi )=\frac{dq(\varphi )}{dt}=\alpha +3\beta \varphi^{2}$$ (6)

where *α*, *β* are parameters associated with the memristor. Magnetic flux *φ* is used as a new variable in the improved dynamical system, and the memristor-coupled-Josephson Junction system is described by

$$\left\{ \begin{array}{l} {\dot{x}}_{1}=x_{2}\\ {\dot{x}}_{2}=(i-g(x_{2})x_{2}-\sin x_{1}-x_{3})/\beta_{C}-k\rho (\varphi )x_{2}\\ {\dot{x}}_{3}=(x_{2}-x_{3})/\beta_{L}\\ \dot{\varphi }=kx_{2} \end{array}\right.;$$ (7)

where the term *kx*_2_ describes the effect of electromagnetic induction, and the term *kρ*(*φ*)*x*_2_ represents the induction current as follows

$$i^{\prime }=\frac{dq}{dt}=\frac{dq}{d\varphi }\frac{d\varphi }{dt}=\rho (\varphi )V=k\rho (\varphi )x_{2}$$ (8)

As a result, there are five bifurcation parameters (*β*_C_, *β*_L_, *k*, *α*, *β*) that can be adjusted to change the outputs and dynamical behaviors in Eq (7) except the input current. Make a contrast between Eq (4) and Eq (7), it is found that the modulation can keep certain symmetry in absence of external forcing as *i* = 0 by using the following transformation

$$\left(x_{1},x_{2},x_{3},\varphi )↔(-x_{1},-x_{2},-x_{3},-\varphi \right)$$ (9)

And the phase constraint is approached by

$$\nabla V=\frac{\partial {\dot{x}}_{1}}{\partial x_{1}}+\frac{\partial {\dot{x}}_{2}}{\partial x_{2}}+\frac{\partial {\dot{x}}_{3}}{\partial x_{3}}+\frac{\partial \dot{\varphi }}{\partial \varphi }=-g(x_{2})-k\rho (\varphi )-1/\beta_{L}$$ (10)

As shown in Eq (5), −*g*(*x*_2_) is always negative. As a result, the improved dynamical system shown in Eq (7) can be dissipative and it is consistent with the original dynamical system shown in Eq (4). In fact, negative dissipativity for dynamical system in the sense (10) may admits unbounded attractors because of existence of Jerk function. For better understanding the dissipativity in the sense of Levinsov (i.e. existence of convex absorbing set), readers can find useful guidance in Refs.[77,78]. In a dynamical view, chaos and even hyperchaos could be induced in Eq (7) by setting appropriate parameters. However, the attractor formation and orbit selection could be also dependent on the selection of initials setting when memristor coupling is used to control the RCL-shunted Junction circuit. Therefore, the outputs voltage *x*_2_ (or *V*) can be adjusted by appropriate gain *k* on the induction current contributed by a memristor, and the coupled circuit is shown as follows

**Fig 2:**
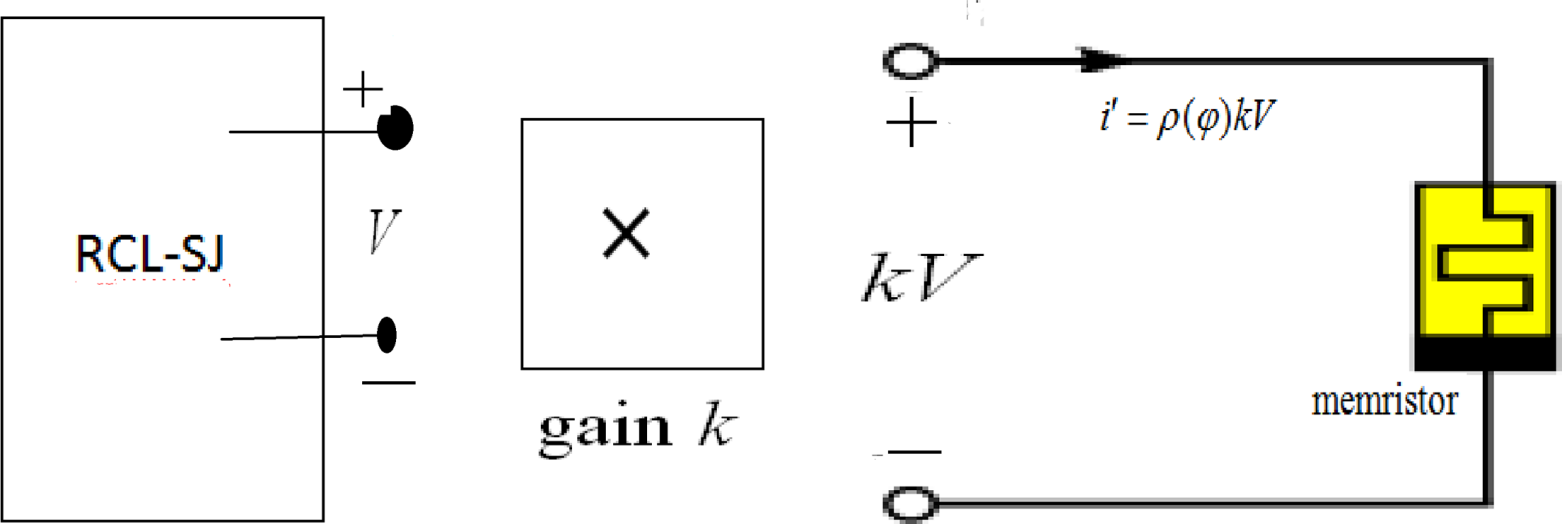
Schematic diagram for memristor-coupled RCL-SJ circuit.

According to Eq (7) and Fig 2, switch the feedback type (negative or positive) can suppress or enhance the chaotic behaviors in RCL-shunted circuit. In the next section, the Lyapunov exponents and phase portraits will be calculated for discussion and suggestion.

## Numerical and experimental investigation

In this section, the fourth order Runge-Kutta algorithm is used to find solutions for Eq (7) with time step *h* = 0.01. For simplicity, *β*_C_ = 0.707, *β*_L_ = 2.6, and parameters *k*, *α*, *β* are carefully selected to investigate the electrical activities and dynamical behaviors. Furthermore, the Lyapunov exponent spectrum is calculated by changing the feedback gain *k*, and the effect of initial setting is also discussed. It is found that the improved Eq (7) can be reduced to the original Eq (4) by setting the coupling intensity as *k* = 0. That is to say, the effect of memristor is critical to induce transition of electrical activities from the outputs. For simplicity, at first, parameters *α*, *β* in the memristor are selected as *α* = 4, *β =* 0.01, the external *DC* current *i* = 1.2. The phase portraits are calculated by setting different feedback gains, and the initial values are selected as (*x*_1_(0), *x*_2_(0), *x*_3_(0), *φ*_0_) = (0.1, 0.2, 0.3, 0).

**Fig 3:**
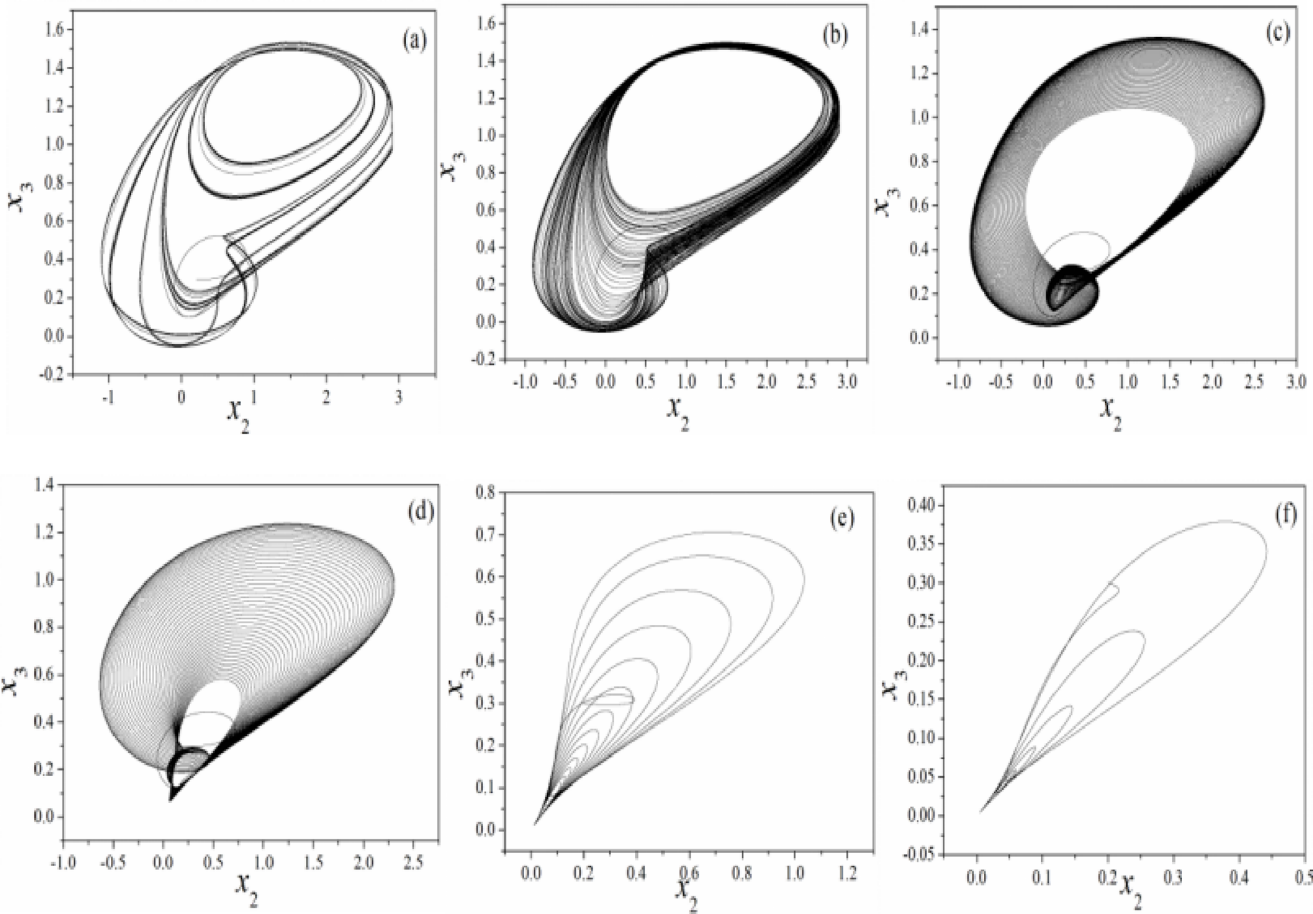
Phase portrait for variable (*x*_2_ *vs*. *x*_3_) is calculated by setting different feedback gains. (a)*k* = 0, (b)*k* = 0.01, (c)*k* = 0.05, (d)*k* = 0.1, (e)*k* = 0.5, (*f*)*k* = 1.2. The parameters in memristor are selected as *α* = 4, *β =* 0.01, and calculating period is within 1500 time units.

**Fig 4:**
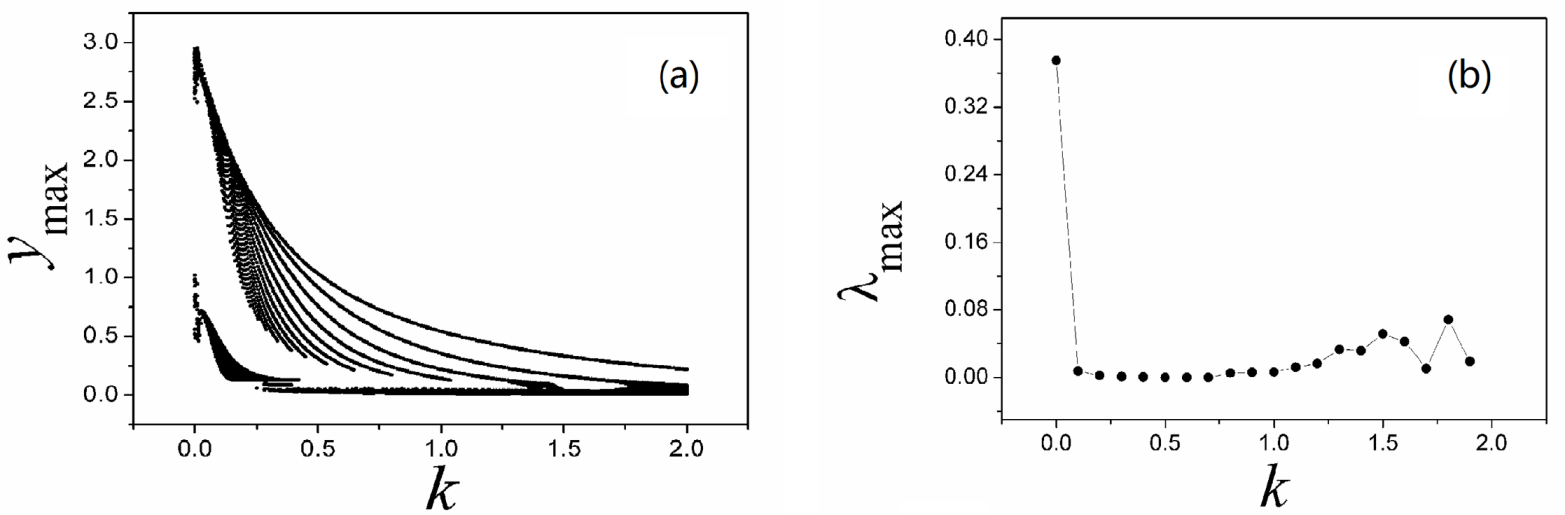
**Bifurcation diagram (a) and Largest Lyapunov diagram (b) are calculated by changing the coefficient *k***. The parameters are selected as *α* = 4, *β =* 0.01, *i* = 1.2, *y*_max_ is the maximal value of sampled time series for variable *y*.

It is found that the chaotic states are suppressed by further increasing the feedback gain *k* for memristor coupled by Josephson junction. According to Eq (4), the memristor can impose negative feedback on the junction voltage and thus the chaos can be controlled by setting larger feedback gains. However, the results in Fig 3B find that the orbits become more dense than the original RCLSJ shown in Eq (1) due to higher nonlinearity when the circuit is modulated by a memristor. Furthermore, the bifurcation analysis and the largest Lyapunov exponent are carried out in Fig 4.

**Fig 5:**
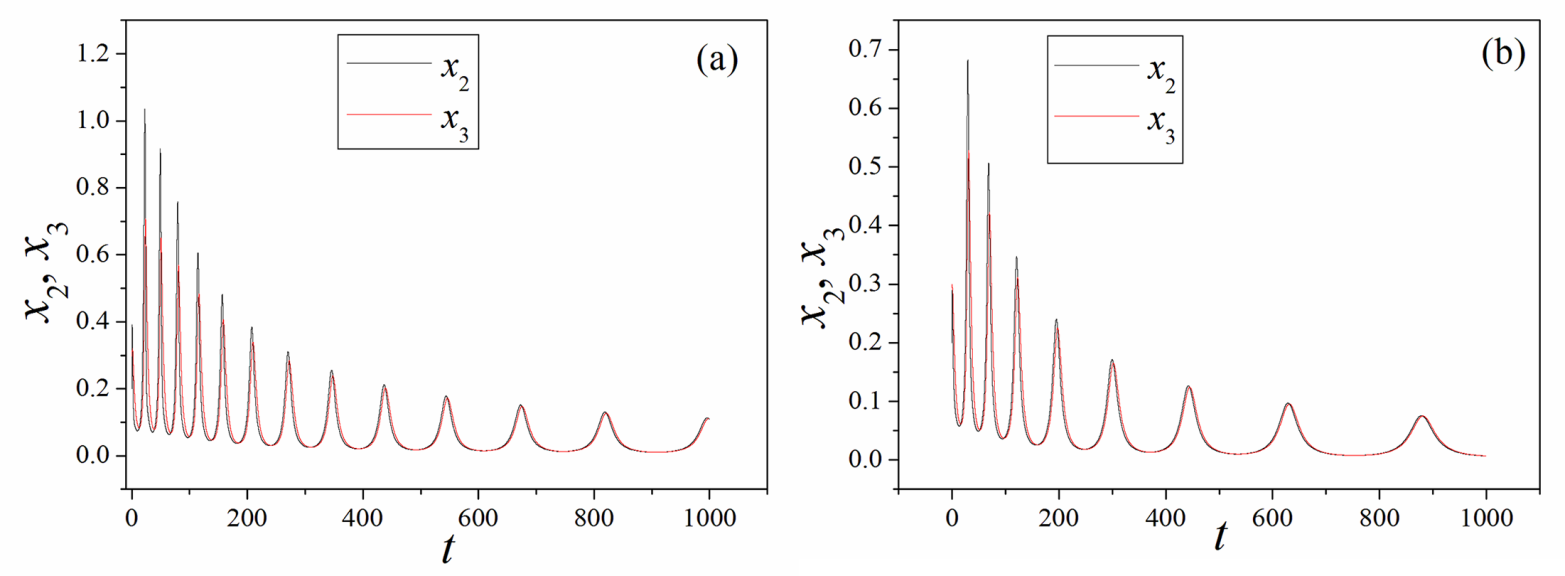
Sampled time series for junction voltage (*x*_2_) and current (*x*_3_). (a)*k* = 0.5, (b)*k* = 0.8. Parameters in memristor are selected as *α* = 4, *β =* 0.01, *i* = 1.2.

The results in Fig 4 confirmed that the largest Lyapunov exponent and the maximal value for variable are decreased synchronously, the orbits become spare before reaching periodical states. In fact, in case of DC stimuli, it is more important to find spiking behavior by setting appropriate feedback gain, and the results are plotted in Fig 5.

**Fig 6:**
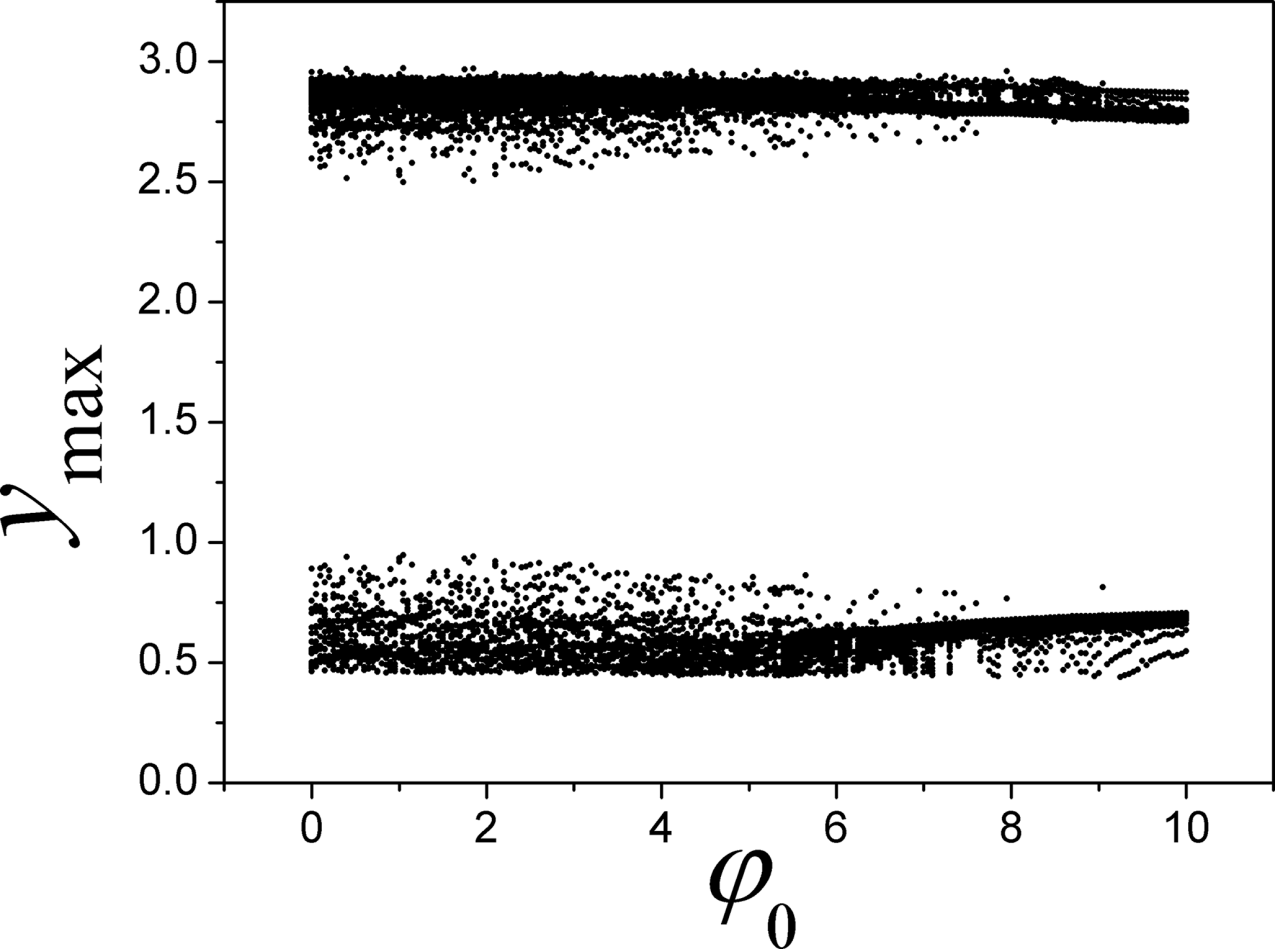
Bifurcation diagram is calculated for maximal state variable *y*_max_ vs the parameter *φ*_0_. The parameters are selected as *α* = 4, *β =* 0.01, *k* = 0.01.

It is found in Fig 5 that the amplitude of junction voltage and current can be decreased by further increasing the feedback gain for memristor, and transient periodical oscillation can be observed in the sampled time series during the control of chaotic behaviors. Extensive numerical results are carried out by setting different initial values for variable *φ*. In Fig 6, the dependence of initial setting for magnetic flux in Eq (7) is calculated.

It is confirmed that the outputs and attractors are much dependent on the initial setting, as a result, switch and resetting the initial value for magnetic flux can trigger different attractors even other parameters are fixed.

**Fig 7:**
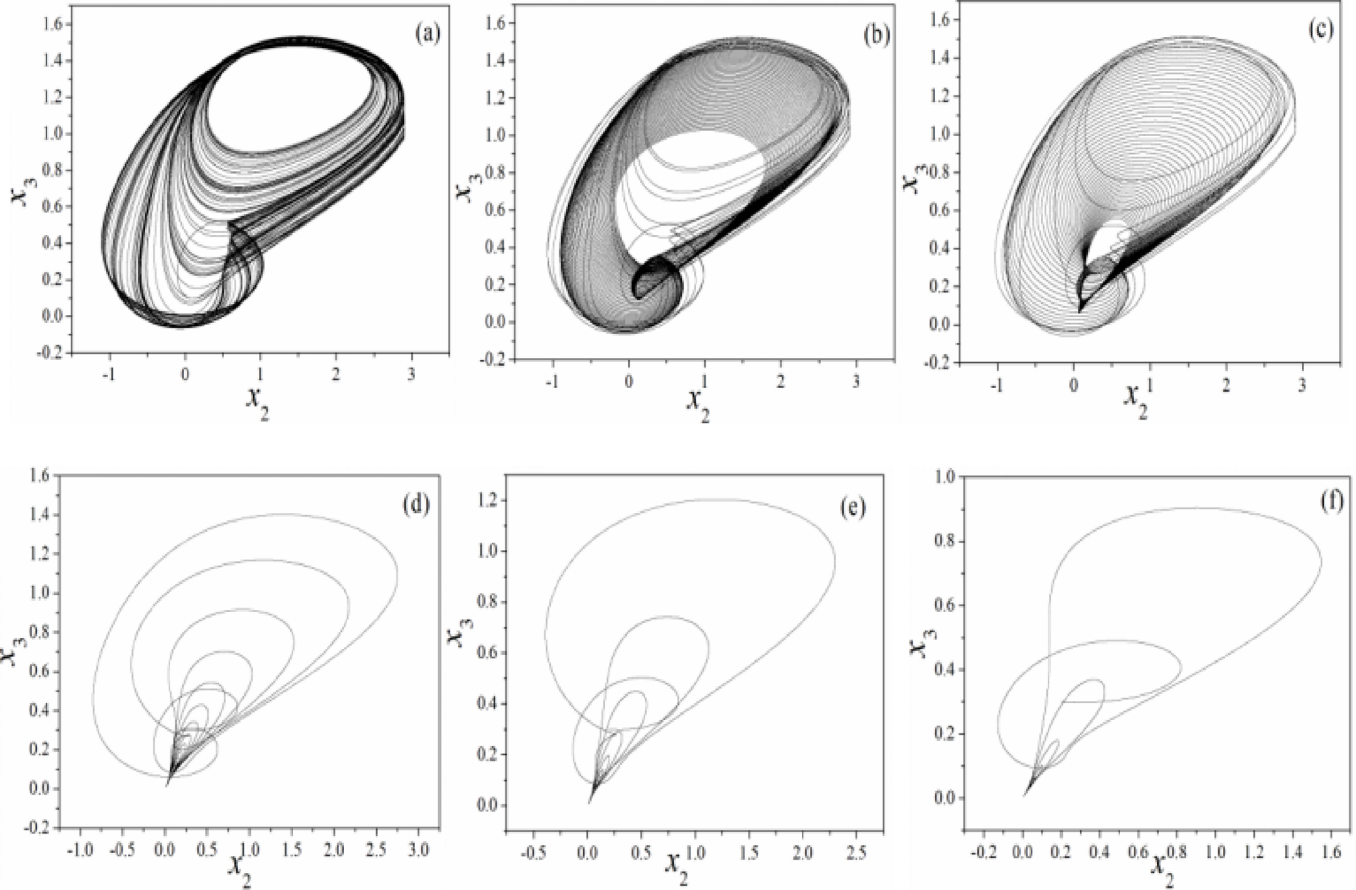
Phase portrait for variable (*x*_2_ *vs*. *x*_3_) is calculated by setting different feedback gain within 1500 time units. (a)*k* = 0.01, (b)*k* = 0.05, (c)*k* = 0.1, (d)*k* = 0.5, (e)*k* = 0.8, (*f*)*k* = 1.2. Parameters in memristor are selected as *α* = 0.1, *β =* 0.01.

However, in the case of negative feedback type, the results seem to be independent of the initials setting completely, and it is some different from the initial-dependent nonlinear system that the developed attractors are much dependent on the initial setting [79]. The potential mechanism could be that the magnetic flux is mainly dependent on junction voltage while it is independent of other variables. In fact, the dynamical behaviors in Eq (7) are also dependent on the external DC stimuli. Therefore, the Lyapunov exponent spectrum is calculated by changing the DC stimuli, and then the feedback gain is also changed to detect the transition of electrical activities at fixed DC stimuli. Furthermore, another group of parameters are setting to investigate the sampled time series and transition of attractors, the results are plotted in Fig 7.

**Fig 8:**
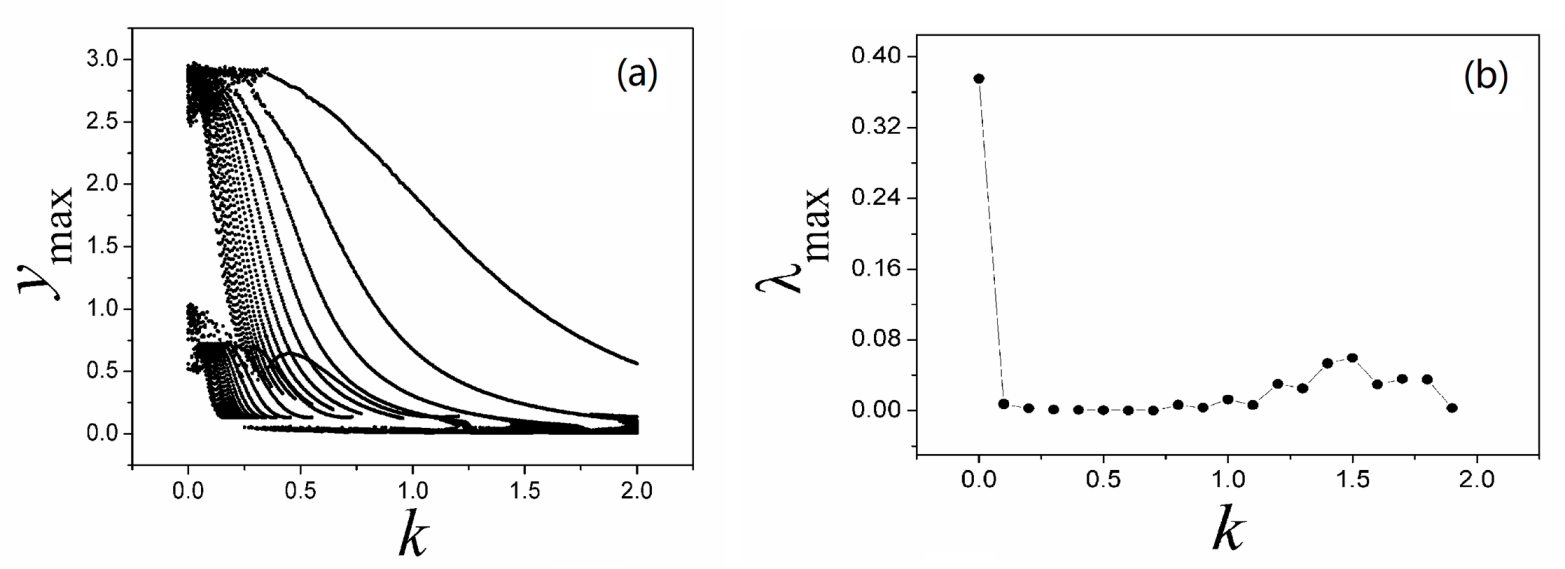
**Bifurcation diagram (a) and Largest Lyapunov diagram (b) are calculated by changing the coefficient *k***. The parameters are selected as *α* = 0.1, *β =* 0.01, *i* = 1.2, *y*_max_ is the maximal value of sampled time series for variable *y*.

With a contrast the results in Fig 3 and Fig 7, it is found that the formed attractors are dependent on the parameters in memristor, and a larger parameter setting for *α* can be more effective to suppress chaos and generate periodical behaviors in the outputs. Also, the bifurcation analysis and largerst Lyapunov exponent are further calculated for verification, and the results are shown in Fig 8.

**Fig 9:**
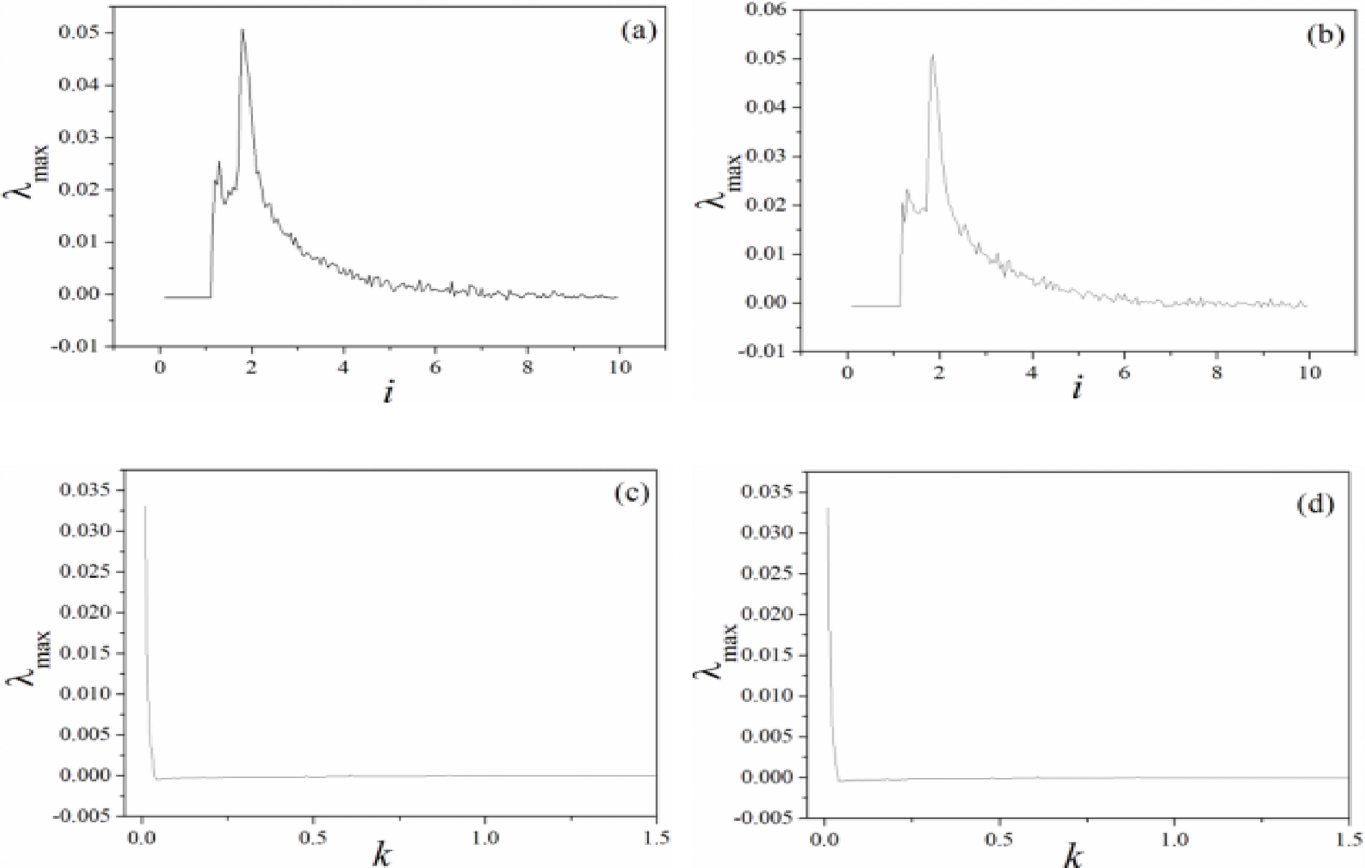
Distribution for largest Lyapunov exponent spectrum is calculated by changing the bifurcation parameter. (a)*α* = 0.1, *β =* 0.01, *k* = 0.01; b)*α* = 4, *β =* 0.01, *k* = 0.01; c)*α* = 0.1, *β =* 0.01, *i* = 1.2; c)*α* = 0.1, *β =* 0.01, *i* = 2.0.

Smaller value for parameters *α*, *β* can decrease the effect of induction current, while increase of feedback intensity *k* can enhance the negative feedback contribution, as a result, the dynamical behaviors are suppressed. The results in Fig 8 found that the largest Lyapunov exponent and maximal value for variables are decreased with increasing the feedback gain. It is also important to investigate the case when external stimuli and also feedback gain *k* are set under a larger value, and the distribution for Lyapunov exponent spectrum is calculated in Fig 9.

**Fig 10:**
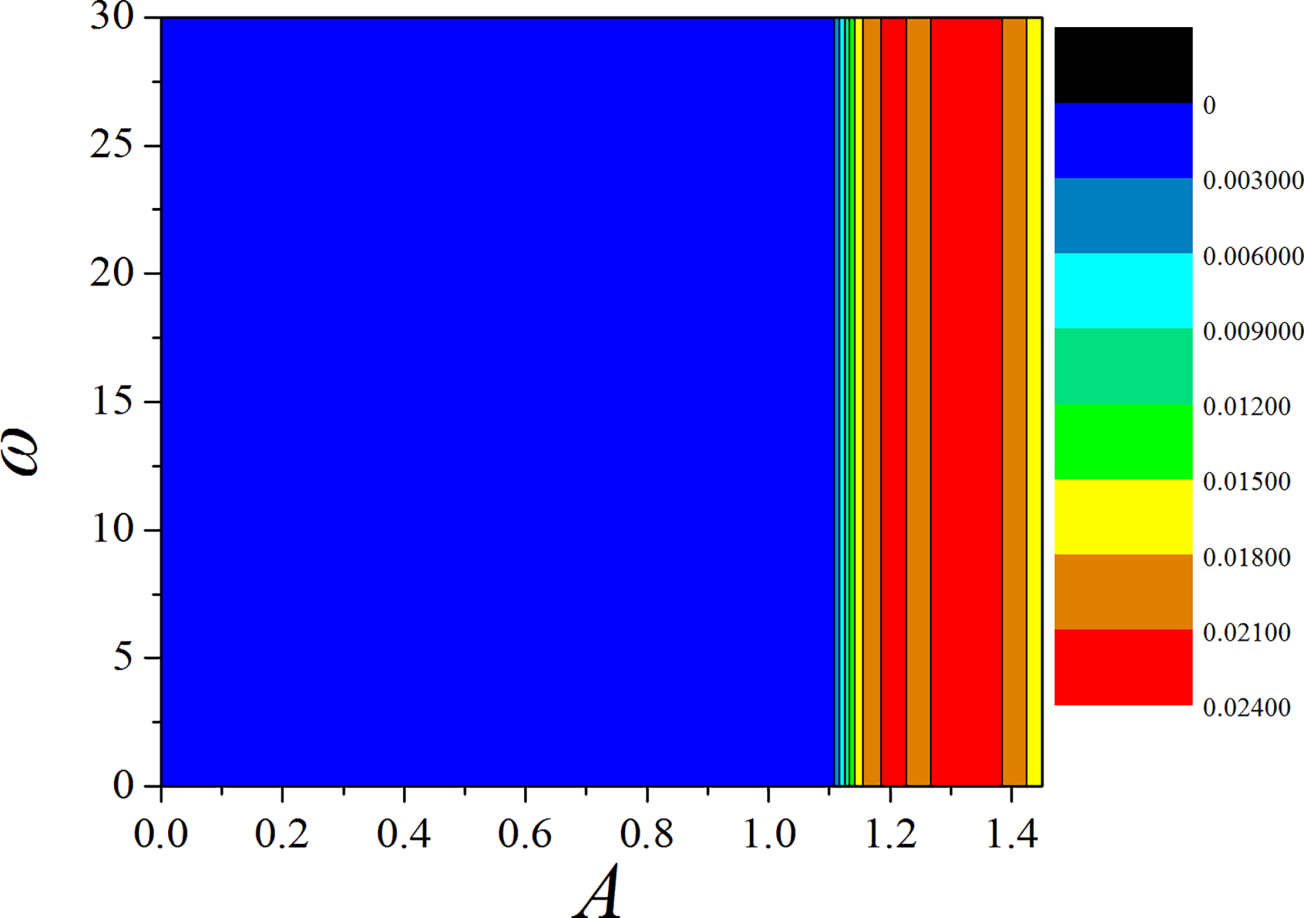
Distribution for largest Lyapunov exponent spectrum is calculated by applying periodical stimuli as *i* = *A*cos*ωt*, *k* = 0.01. The snapshots are plotted in color scale.

The results in Fig 9 found that appropriate external stimuli is helpful to support the emergence of chaos, then the chaos is removed by further increasing the external DC stimuli. When the effect of memristor is enhanced by increasing the feedback gain *k*, the chaotic behavior can be suppressed and even removed. The potential mechanism is that larger coupling intensity between memristor and RCL-shunted junction can input larger forcing current into the junction that chaos can be controlled completely. In Fig 10, the largest Lyapunov exponent spectrum is calculated by applying different periodical stimuli on the junction.

It is confirmed that chaos can be induced by appropriate periodical stimuli when the feedback on memristor is weak. By further increasing the feedback gain in memristor, the chaos can be suppressed completely. It is important to investigate the same case on the chaotic Jerk circuit [80,81] composed of multi-scroll attractors. The dynamical equations are described by

$$\left\{ \begin{array}{l} \dot{x}=y\\ \dot{y}=z\\ \dot{z}=-ay-az+a\sin2\pi bx \end{array}\right.;$$ (11)

**Fig 11:**
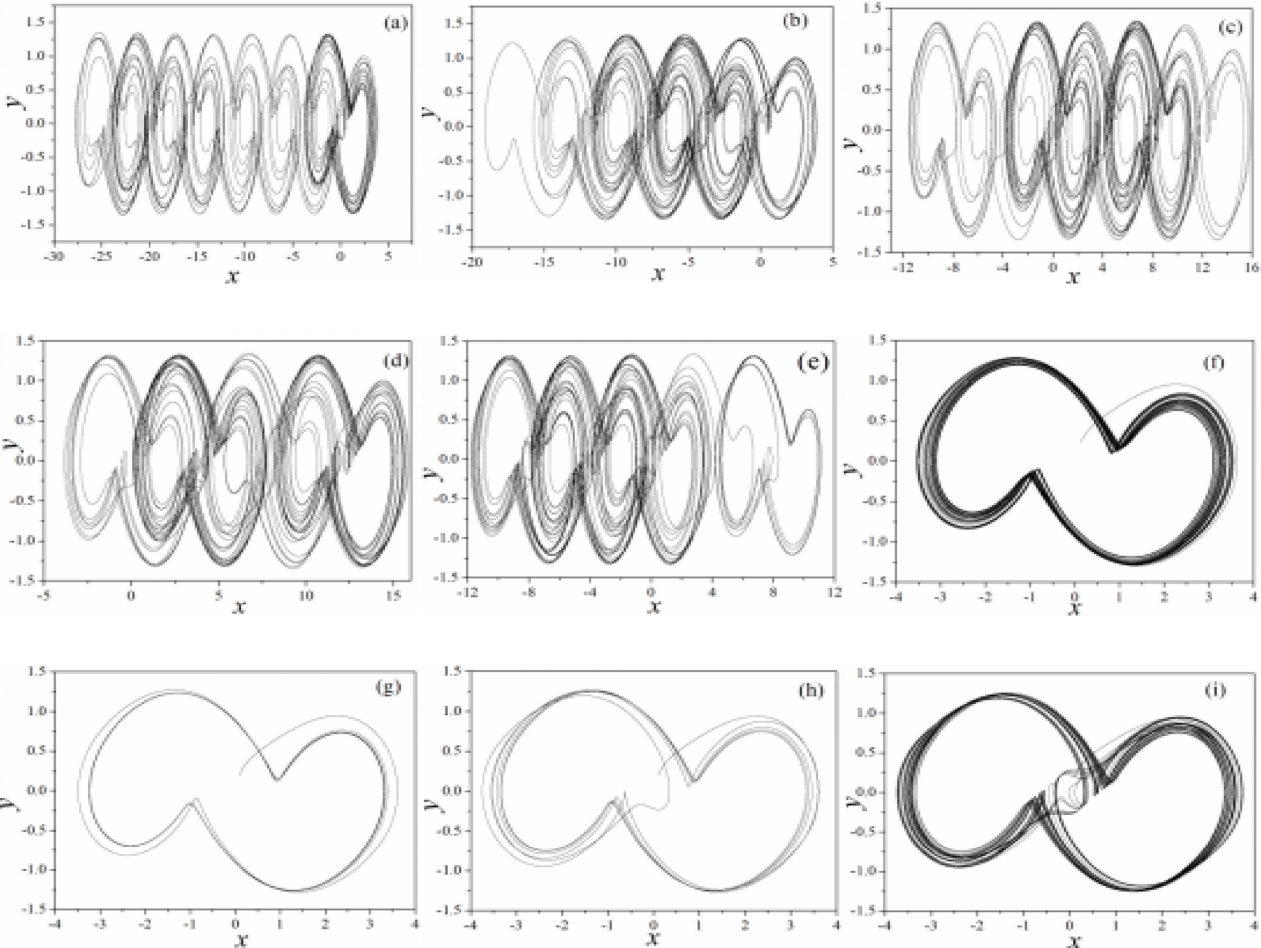
Selection of multi-attractor in the memristor-coupled-Jerk circuit. (a)*k* = 0, (b)*k* = 0.01, (c) *k* = 0.02, (d)*k* = 0.05, (e)*k* = 0.1, (f)*k* = 0.2, (g)*k* = 0.3, (h)*k* = 0.4, (i)*k* = 0.5. Parameters are selected as *ρ*(*φ*) = 0.1+0.03*φ*^*2*^, *a* = 0.3, b = 0.25 and the calculating time is 1200 time units. The modulation is realized on the third variable.

As confirmed in Ref.[82], the Jerk circuit shown in Eq (11) can find chaotic behavior by setting *a* = 0.3, b = 0.25, and the number of scroll attractors is dependent on the calculating time. As a result, more scroll attractors can be generated by increasing the calculating time. In Ref.[82], Heaviside function is used to control the number selection of scroll attractors, and the result was also verified on the PSpice tool. With the similar scheme mentioned above, the memristor-coupled Jerk circuit can be described by

$$\left\{ \begin{array}{l} \dot{x}=y\\ \dot{y}=z\\ \dot{z}=-ay-az+a\sin2\pi bx-k\rho (\varphi )z\\ \dot{\varphi }=kz \end{array}\right.;$$ (12)

where the term *ρ*(*φ*) represents the same meaning in Eq (6), as a result, the Eq (12) is reduced to the Jerk circuit as shown in Eq (11) when the effect of memristor is removed. To be consistent with the previous assumption, parameters are selected as *a* = 0.3, *b* = 0.25, and the feedback gain *k* is changed to stabilize the multi-scroll attractors. According to Eq (12), multi-scroll attractors are calculated in Fig 11 by selecting appropriate parameter *k*.

**Fig 12:**
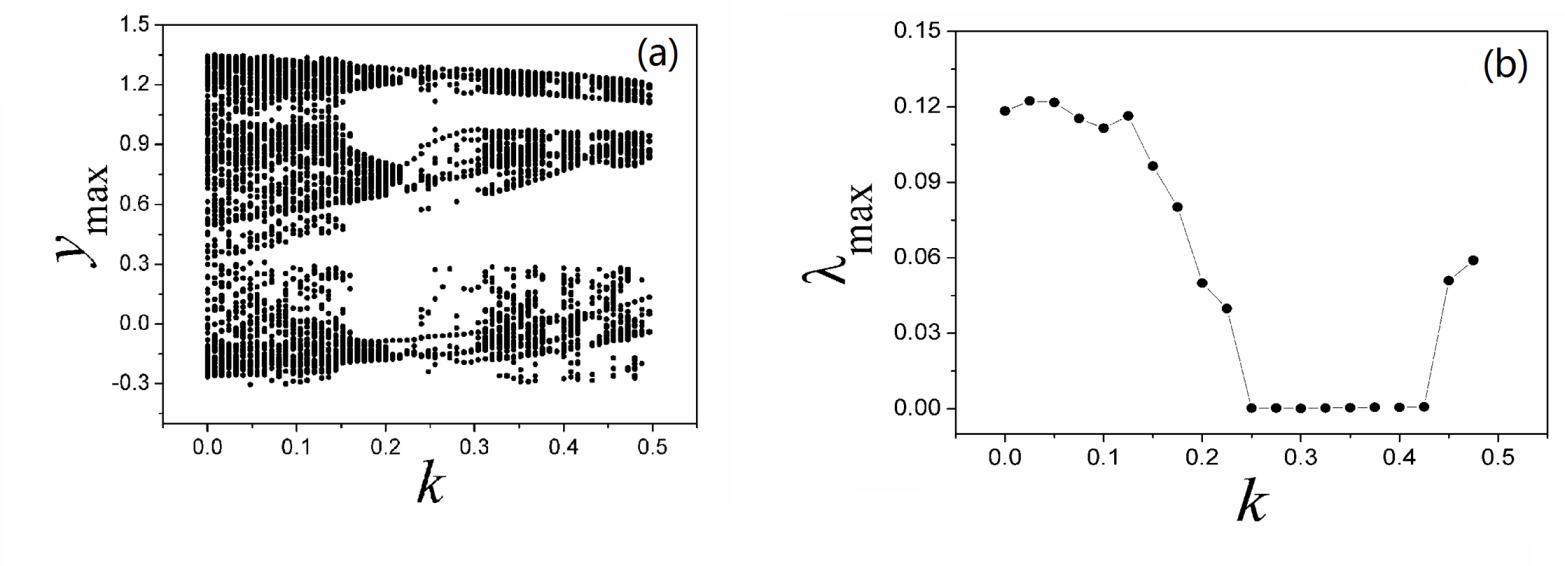
**Bifurcation diagram (a) and Largest Lyapunov diagram (b) are calculated by changing the coefficient *k***. The parameters are selected as *α* = 0.1, *β =* 0.01, *i* = 1.2, *a* = 0.3, *b* = 0.25 for Eq (12). *y*_max_ is the maximal value of sampled time series for variable *y*.

It is confirmed that different numbers of scroll attractors can be selected by applying appropriate feedback gain in memristor, and extensive numerical investigations find that appropriate feedback gain can be found to select the required number of scroll attractors even the calculating period is increased. With the similar scheme, sampled time series are calculated to find the largest Lyapunov exponent and bifurcation stability, the results are shown in Fig 12.

**Fig 13:**
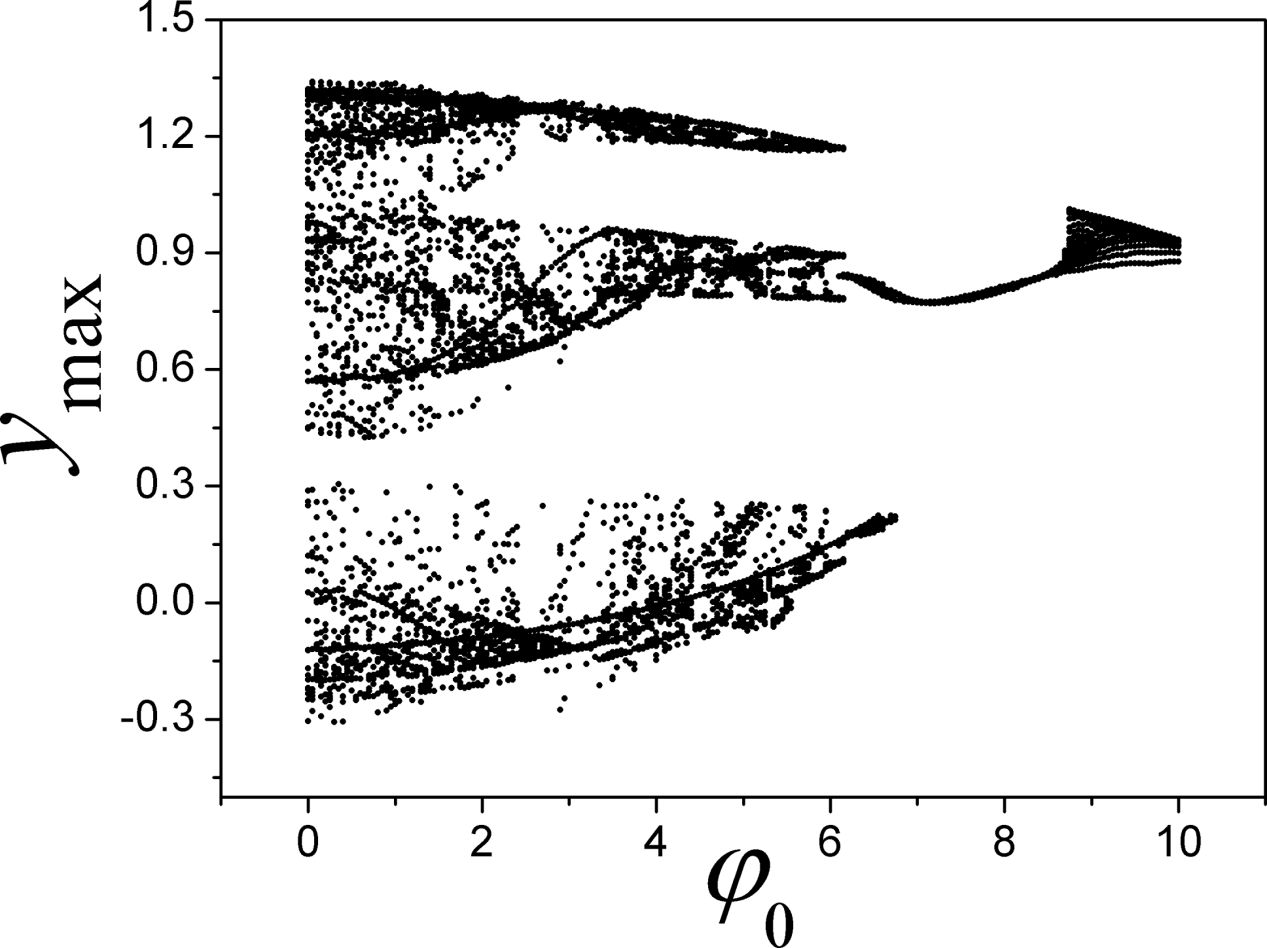
Bifurcation diagram is calculated for maximal state variable *y*_max_ vs the parameter *φ*_0_. The parameters are selected as *α* = 0.1, *β =* 0.01, *a* = 0.3, *b* = 0.25, *k* = 0.1 for Eq (12).

Compared the results in Fig 12 with Fig 8, the bifurcation diagram becomes more abundant and the evolution of largest Lyapunov exponent shows distinct transmission to predict the occurrence of chaos to periodicity to chaos. As presented in Eq (12), memristor function and magnetic variable are used, and the dynamical behaviors could be dependent on the initial setting as well. In Fig 13, the dependence of initial setting is detected.

**Fig 14:**
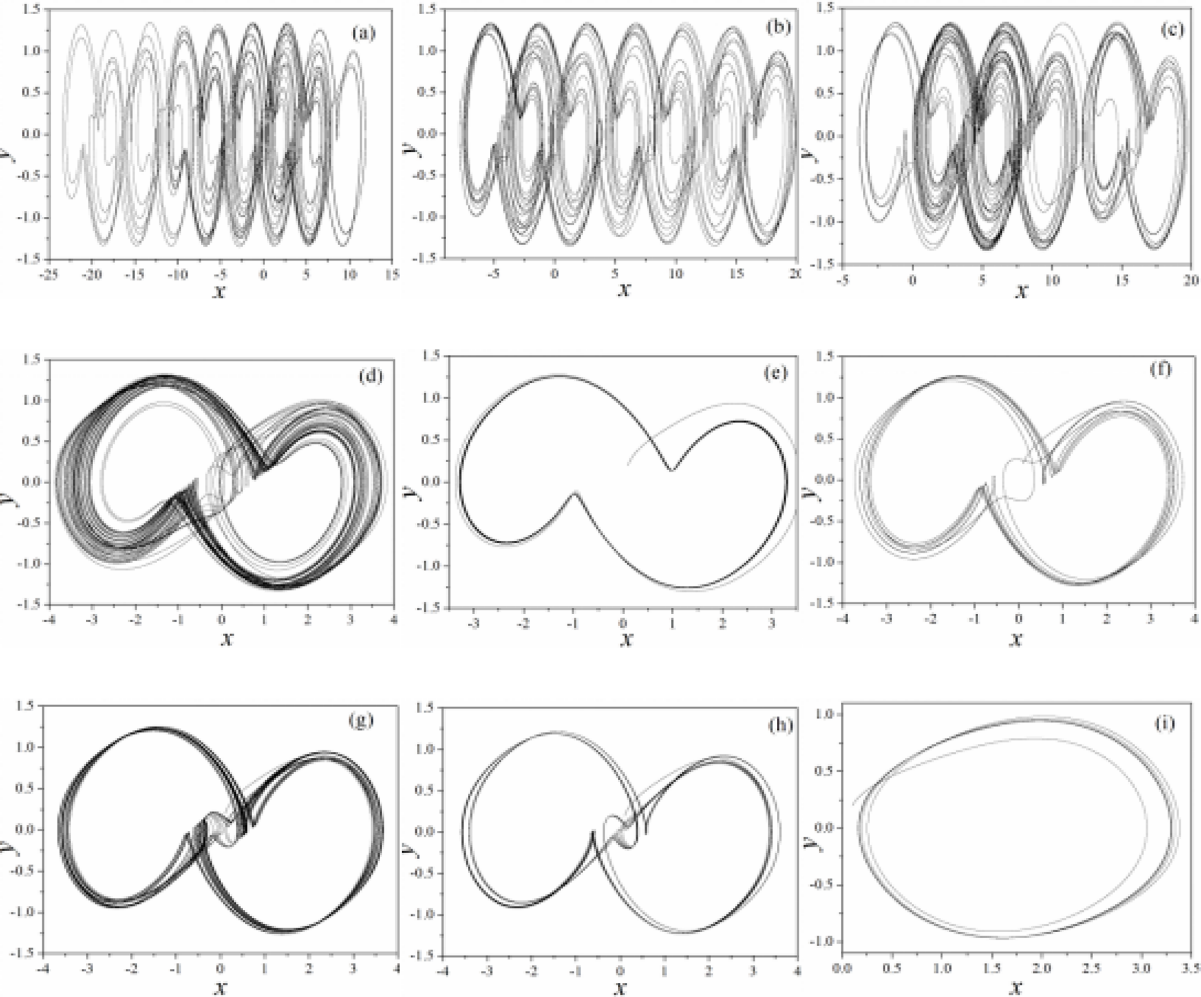
Selection of multi-attractor in the memristor-coupled-Jerk circuit. (a)*k* = 0.01, (b)*k* = 0.02, (c) *k* = 0.05, (d)*k* = 0.1, (e)*k* = 0.2, (f)*k* = 0.3, (g)*k* = 0.4, (h)*k* = 0.5, (i)*k* = 1.0. Parameters are selected as *ρ*(*φ*) = 0.1+0.03*φ*^*2*^, *a* = 0.3, b = 0.25 and the calculating time is 1200 time units. The modulation is realized on the second variable.

The results in Fig 13 confirmed that the improved Jerk circuit shown in Eq (12) is also much dependent on the initial values, and it is helpful to trigger and form different attractors by resetting the initial values for magnetic flux. We also investigated the case when memristor modulation is applied on the second variable, it reads as follows

$$\left\{ \begin{array}{l} \dot{x}=y\\ \dot{y}=z-k\rho (\varphi )y\\ \dot{z}=-ay-az+a\sin2\pi bx\\ \dot{\varphi }=ky \end{array}\right.;$$ (13)

where the parameters are used the same for Eq (12), the results are calculated in Fig 14.

**Fig 15:**
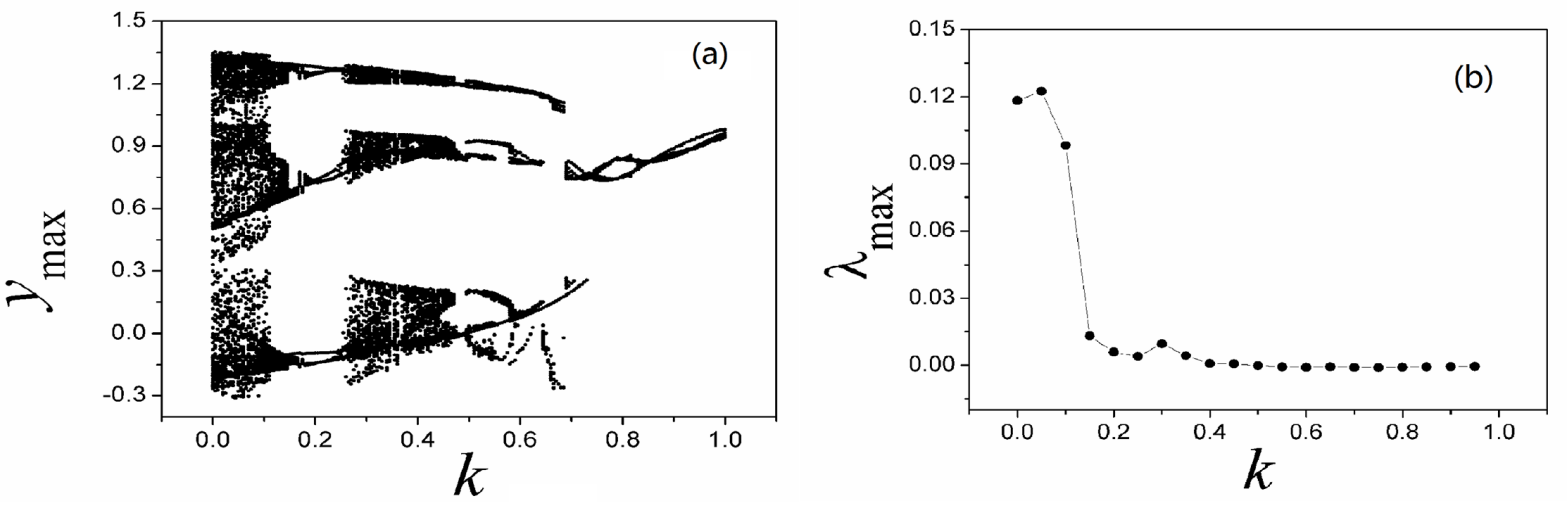
**Bifurcation diagram (a) and Largest Lyapunov diagram (b) are calculated by changing the coefficient *k***. The parameters are selected as *α* = 0.1, *β =* 0.01, *i* = 1.2, *a* = 0.3, *b* = 0.25 for Eq (13). *y*_max_ is the maximal value of sampled time series for variable *y*.

As confirmed in Fig 14, the multi-scroll attractors can be stabilized from infinite number of scroll attractors by applying appropriate feedback on the Jerk circuit. That is to say, the memristor coupling on the Jerk circuit is effective to control the multi-scroll attractors completely. Furthermore, the bifurcation analysis and initials dependence are calculated. In Fig 15, sampled time series for variable are produced to find bifurcation transition and the largest Lyapunov exponent for Eq (13).

**Fig 16:**
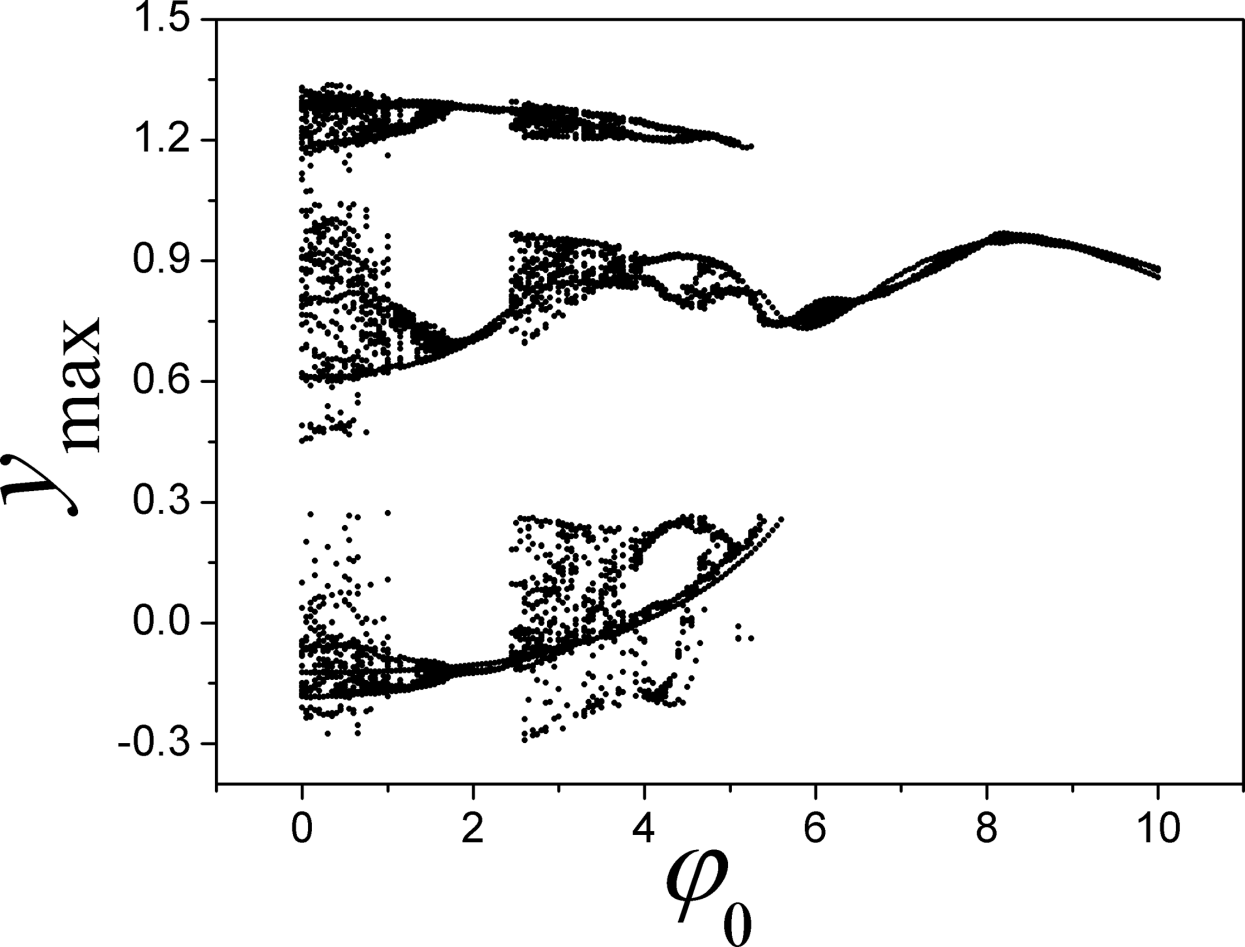
Bifurcation diagram is calculated for maximal state variable *y*_max_ vs the parameter *φ*_0_. The parameters are selected as *α* = 0.1, *β =* 0.01, *a* = 0.3, *b* = 0.25, *k* = 0.1 for Eq (13).

As confirmed in Fig 15, the maximal value for the variable is modulated greatly and the bifurcation sounds like anti-double periodical bifurcation with increasing the feedback coefficient *k*, and the largest Lyapunov exponent is decreased greatly. As a result, continuous decreasing for a larger feedback coefficient *k* could trigger double periodical bifurcation and chaos is generated. We also checked the initials dependence for Eq (13), and the results are presented in Fig 16.

That is, the outputs and the attractors are much dependent on the initial values for variable magnetic flux. As a result, it provides an effective way to select appropriate attractor and orbits by setting appropriate initial values for variable magnetic flux.

In fact, the modulation from memristor can impose nonlinear negative feedback on the dynamical system, thus the outputs can be adjusted carefully. This scheme can be further used to control chaos, hyperchaos in other dynamical systems. For readers’ interests, these results can be further verified on PSpice and experimental circuits. It is also important to clarify some relevant questions for dynamical analysis. The algorithm proposed by Wolf [83] could be effective to estimate the possibility for occurrence of chaos by calculating the Lyapunov exponents with long period series and positive Lyapunov exponent seems to be effective to give evidence for emergence of chaos for most of dynamical systems. Maybe, finite-time local Lyapunov exponents spectrum and finite-time local Lyapunov exponent could be more appreciated since LEs were computed along one considered trajectory and for a finite-time. If LEs are the same for any initial point in the phase space, then Kaplan and Yorke suggested to call them “absolute” (see Frederickson et all 1983, page 190) and wrote that such absolute values rarely exist (thus a local LEs computed along one specific trajectory may not characterize the behaviour of the system for all initial data, especially in the case of multistability. For example, when memristor is considered, the effect of initial setting becomes more important. Therefore, the dynamical analysis for Eq (6) is worthy of further investigating, and readers can find useful scheme guidance in Refs.[84,85].

## Conclusions

In this paper, memristor is used to control the chaotic behaviors in a RCL-shunted Junction circuit by imposing induction current generated from memristor. The extended and improved four-variable dynamical system can be controlled by the coupling intensity (or feedback gain) between the memristor and the RCL-shunted junction circuit. The effect of external stimuli is discussed as well. Furthermore, this problem is also verified to control multi-scroll attractors in jerk circuit, and it is found that finite number of attractors can be stabilized from the infinite number of scroll-attractors by applying appropriate feedback gain in memristor coupled with the jerk circuit. As a result, this scheme can be further used to suppress (or enhance, positive feedback type) chaotic behaviors in other nonlinear dynamical systems driven by memristor, which is also effective to bridge two neurons and then the synchronization approach can be controlled[86–88].

## Supporting information

S1 File The data for contour plot of largest Lyapunov exponent spectrum(shown in Fig 10). (DAT)
